# Supplementary material for: One-step direct oxidation of fullerene-fused alkoxy ethers to ketones for evaporable fullerene derivatives
Source: Commun Chem. 2021 May 21;4:74. doi: 10.1038/s42004-021-00511-4 (PMC9814674; doi:10.1038/s42004-021-00511-4)
Supplement: Supplementary file 1 — Supplementary Information [file 42004_2021_511_MOESM1_ESM.pdf]

Supplementary Information

One-step direct oxidation of fullerene-fused alkoxy ethers to ketones for evaporable fullerene derivatives

Hao-Sheng Lin,[1,5,6] Yue Ma,[2,6] Rong Xiang,[1] Sergei Manzhos,[3] Il Jeon,[1,4] Shigeo Maruyama[1] and Yutaka Matsuo[1,2,5]*

[1] Department of Mechanical Engineering, School of Engineering, The University of Tokyo, 7-3-1 Hongo, Bunkyo-ku, Tokyo 113-8656, Japan.

[2] Hefei National Laboratory for Physical Sciences at the Microscale, School of Chemistry and Materials of Science, University of Science and Technology of China, 96 Jinzhai Road, Hefei, Anhui 230026, China

[3] Centre Énergie Matériaux Télécommunications, Institut National de la Recherche Scientifique, 1650 boulevard Lionel-Boulet, Varennes QC J3X1S2 Canada.

[4] Department of Chemistry Education, Graduate School of Chemical Materials, Pusan National University, 63-2 Busandaehak-ro, Busan 46241, Republic of Korea.

[5] Department of Chemical System Engineering, Graduate School of Engineering, Nagoya University, Furo-cho, Chikusa-ku, Nagoya 464-8603, Japan.

[6] These authors contributed equally

Correspondence and requests for materials should be addressed to Yut. M. (matsuo@photon.t.u-tokyo.ac.jp)

1. Isotope-labelling experiments

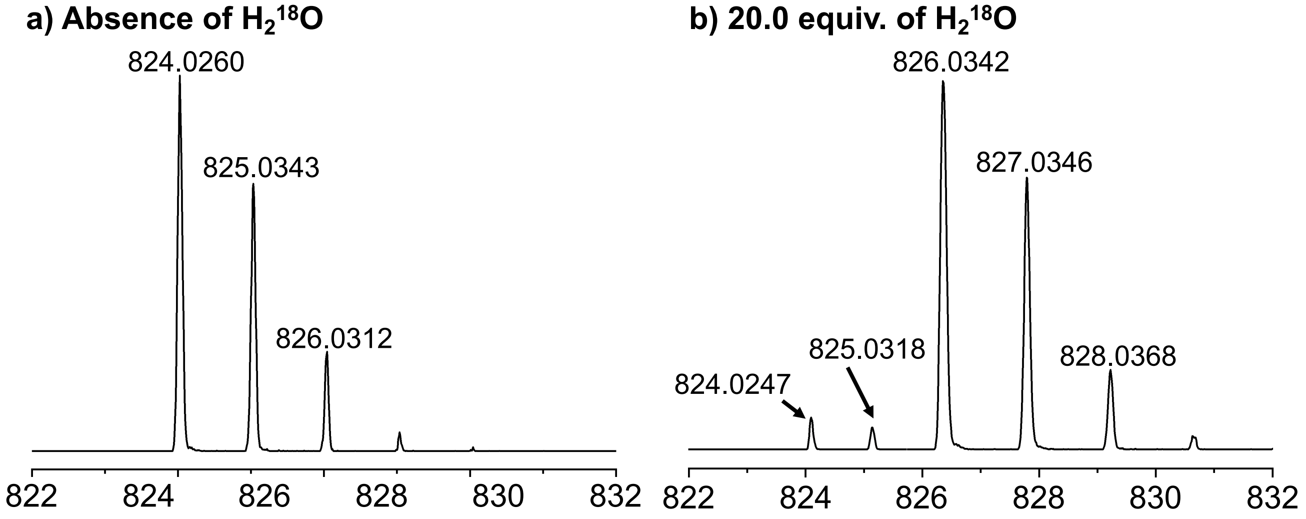

Supplementary Figure 1. ^{18}O isotope-labelling experiments. a) HRMS of non-^{18}O labeled **2a**. **b)** HRMS of ^{18}O labeled **2a-(^{18}O)**.

Supplementary Table 1 HRMS data of non-^{18}O labeled 2a.

Molecular formula	Theoretical *m/z*	Found *m/z*	Absolute intensity (a.u.)	Normalized intensity (%)[a]
$C_{67}H_4O$	824.0262	824.0260	3798[b]	100
	825.0296	825.0343	2705	71
	826.0329	826.0312	1006	26

[a]Calculated from the intensity of each peak divided by the intensity of the base peak. [b]Selected as the base peak.

Supplementary Table 2 HRMS data of ^{18}O labeled 2a-(^{18}O).

Molecular formula	Theoretical *m/z*	Found *m/z*	Absolute intensity (a.u.)	Normalized intensity (%)[a]
$C_{67}H_4O$	824.0262	824.0247	619	9
	825.0329	825.0318	426	6
$C_{67}H_4{}^{18}O$	826.0303	826.0342	7051[b]	100
	827.0337	827.0346	5201	74
	828.0370	828.0368	1526	22

[a]Calculated from the intensity of each peak divided by the intensity of the base peak. [b]Selected as the base peak.

2. Kinetic studies

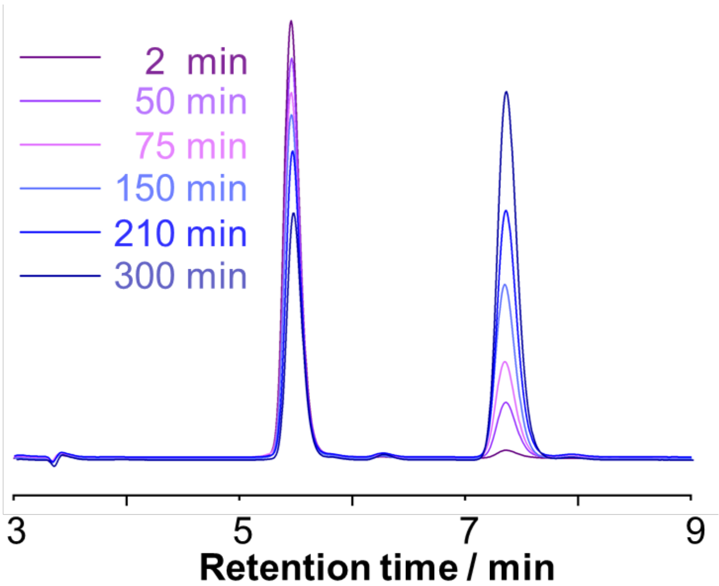

Supplementary Figure 2. HPLC profile of the oxidation of 1a at 353 K.

Supplementary Table 3 HPLC report of the oxidation of 1a at 353 K.

Time (min)	1a			2a		
	Intensity (a.u.)	Conc. (×10^3 mM)	Ratio (%)	Intensity (a.u.)	Conc. (×10^3 mM)	Ratio (%)
5	60503	1.19	99.7	192	0.01	0.3
10	60091	1.18	98.8	737	0.00	1.2
15	48563	1.16	97.1	1444	0.01	2.9
20	33130	1.14	95.5	1548	0.03	4.5
30	46772	1.09	91.7	4217	0.05	8.3
40	45870	1.05	88.4	6006	0.10	11.6
50	38421	1.01	85.1	6732	0.14	14.9
60	37013	0.96	80.8	8773	0.18	19.2
75	31602	0.90	75.9	10014	0.23	24.1
90	24675	0.84	70.7	10224	0.29	29.3
105	25308	0.82	69.3	11234	0.35	30.7
120	27245	0.78	65.1	14578	0.37	34.9
150	32822	0.73	61.3	20744	0.41	38.7
180	29339	0.65	54.2	24780	0.46	45.8
210	33866	0.57	48.0	36742	0.54	52.0
240	29815	0.50	42.4	40444	0.62	57.6
270	25656	0.45	37.5	42672	0.69	62.5
300	22041	0.40	33.2	44321	0.74	66.8

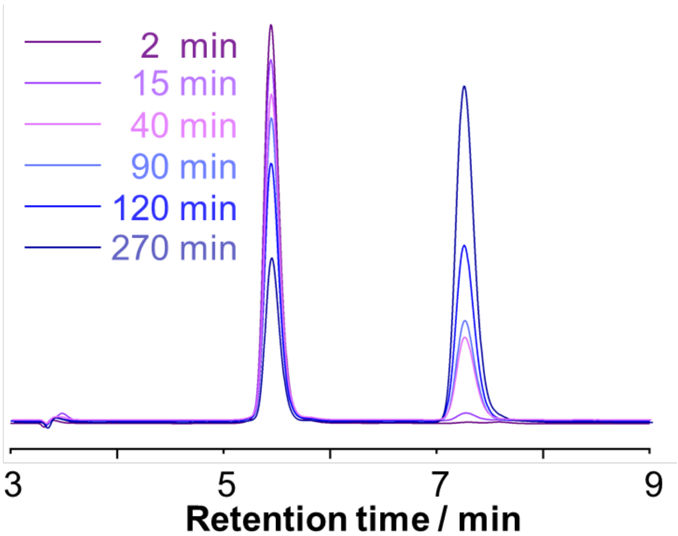

Supplementary Figure 3. HPLC profile of the oxidation of 1a at 360 K.

Supplementary Table 4 HPLC report of the oxidation of 1a at 360 K.

Time (min)	1a			2a		
	Intensity (a.u.)	Conc. (×10^3 mM)	Ratio (%)	Intensity (a.u.)	Conc. (×10^3 mM)	Ratio (%)
2	49142	1.18	99.3	338	0.01	0.7
5	42627	1.18	95.3	2088	0.06	4.7
10	51247	1.15	97.0	1598	0.04	3.0
15	44626	1.09	91.7	3789	0.09	7.8
20	45852	1.06	88.7	5665	0.13	11.0
30	45742	0.97	81.1	10289	0.22	18.2
40	39463	0.89	75.0	12895	0.29	24.5
50	36041	0.81	68.1	16463	0.37	31.1
60	33861	0.74	62.3	20023	0.44	36.9
75	24030	0.64	53.7	20467	0.55	45.8
90	23166	0.55	46.2	26666	0.63	53.2
105	20190	0.48	40.0	30097	0.71	59.6
120	18315	0.41	34.6	34417	0.77	64.9
150	14173	0.33	27.8	36592	0.85	71.8
180	12636	0.27	22.5	43104	0.91	76.7
210	9066	0.23	18.9	38605	0.96	80.7
240	8790	0.23	18.8	37965	0.95	79.9
270	8401	0.20	17.2	40442	0.97	81.2
300	8012	0.20	17.0	39117	0.99	82.8

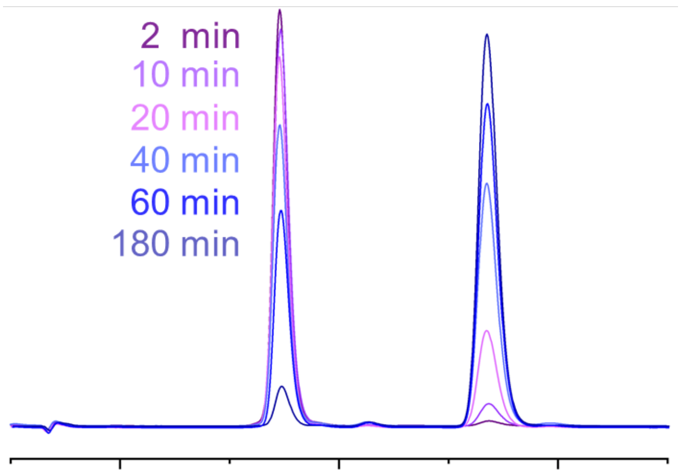

Supplementary Figure 4. HPLC profile of the oxidation of 1a at 368 K.

Supplementary Table 5 HPLC report of the oxidation of 1a at 368 K.

Time (min)	1a			2a		
	Intensity (a.u.)	Conc. (×10^3 mM)	Ratio (%)	Intensity (a.u.)	Conc. (×10^3 mM)	Ratio (%)
2	37335	1.17	98.4	589	0.02	1.6
5	57844	1.16	97.5	1449	0.03	2.4
10	37845	1.11	93.3	2738	0.08	6.7
15	44423	1.01	85.2	7697	0.18	14.8
20	33897	0.89	75.0	11282	0.30	25.0
30	30723	0.72	60.7	19866	0.47	39.3
40	22065	0.58	49.1	22872	0.61	50.9
50	19491	0.48	40.3	28883	0.71	59.7
60	14846	0.41	34.3	28408	0.78	65.7
75	12448	0.32	26.6	34265	0.87	73.3
90	8151	0.23	19.1	34471	0.96	80.9
105	5951	0.18	14.8	34214	1.01	85.2
120	5558	0.14	11.7	42010	1.05	88.3
150	3986	0.11	8.8	41181	1.08	91.2
180	3457	0.09	7.3	43798	1.10	92.7
210	2405	0.08	6.4	35316	1.11	93.6
240	2442	0.07	5.9	39160	1.12	94.1
270	2321	0.06	5.4	40660	1.13	94.6
300	2364	0.06	5.0	44916	1.13	95.0

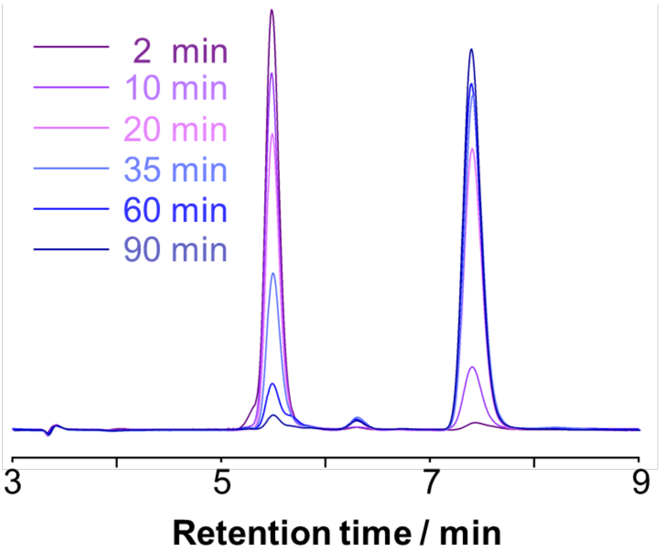

Supplementary Figure 5. HPLC profile of the oxidation of 1a at 375 K.

Supplementary Table 6 HPLC report of the oxidation of 1a at 375 K.

<table>
<tr><th rowspan="2">Time (min)</th><th colspan="3">1a</th><th colspan="3">2a</th></tr>
<tr><th>Intensity (a.u.)</th><th>Conc. (×10^3 mM)</th><th>Ratio (%)</th><th>Intensity (a.u.)</th><th>Conc. (×10^3 mM)</th><th>Ratio (%)</th></tr>
<tr><td>0</td><td>38594</td><td>1.19</td><td>100</td><td>0</td><td>0.00</td><td>0.0</td></tr>
<tr><td>2</td><td>49074</td><td>1.10</td><td>92.1</td><td>3655</td><td>0.08</td><td>6.9</td></tr>
<tr><td>4</td><td>38471</td><td>1.01</td><td>84.8</td><td>6442</td><td>0.17</td><td>14.2</td></tr>
<tr><td>6</td><td>31451</td><td>0.89</td><td>74.4</td><td>10458</td><td>0.29</td><td>24.7</td></tr>
<tr><td>11</td><td>26750</td><td>0.70</td><td>58.7</td><td>18045</td><td>0.47</td><td>39.6</td></tr>
<tr><td>16</td><td>18157</td><td>0.52</td><td>44.1</td><td>22376</td><td>0.65</td><td>54.3</td></tr>
<tr><td>26</td><td>12423</td><td>0.32</td><td>26.7</td><td>33033</td><td>0.84</td><td>70.9</td></tr>
<tr><td>36</td><td>5829</td><td>0.17</td><td>14.6</td><td>34144</td><td>0.99</td><td>83.5</td></tr>
<tr><td>46</td><td>5055</td><td>0.13</td><td>11.0</td><td>40283</td><td>1.04</td><td>87.3</td></tr>
<tr><td>56</td><td>2711</td><td>0.08</td><td>6.6%</td><td>37688</td><td>1.09</td><td>91.8</td></tr>
<tr><td>71</td><td>1382</td><td>0.04</td><td>3.6%</td><td>36153</td><td>1.13</td><td>95.0</td></tr>
<tr><td>86</td><td>1403</td><td>0.04</td><td>3.6%</td><td>37027</td><td>1.13</td><td>94.6</td></tr>
<tr><td>120</td><td>1398</td><td>0.04</td><td>3.5%</td><td>37102</td><td>1.13</td><td>94.7</td></tr>
</table>

3. Mechanistic studies

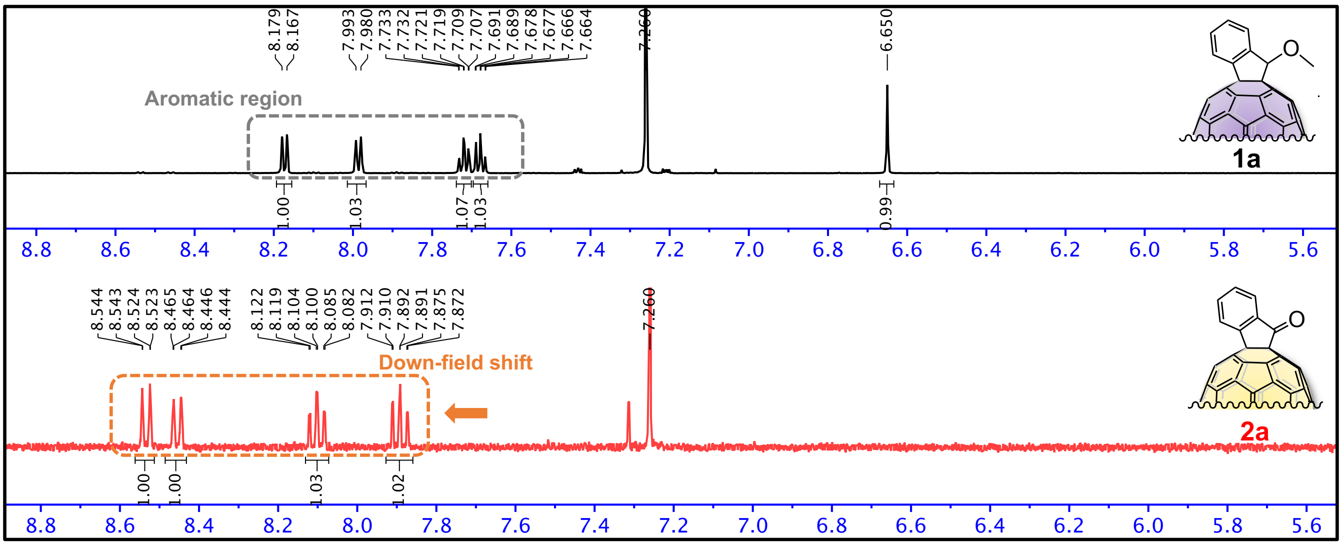

Supplementary Figure 6. The comparison of ^{1}H NMR of 1a and 2a with $CDCl_3$ as internal reference (δ = 7.260 ppm).

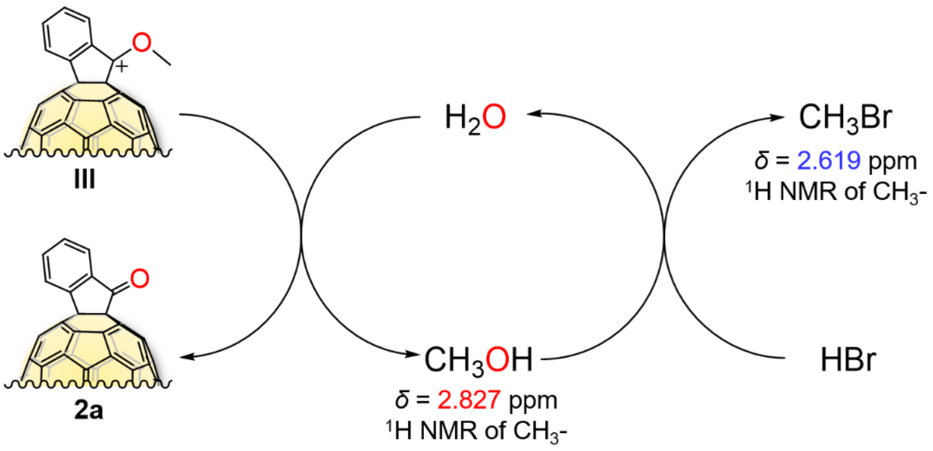

Supplementary Figure 7. Plausible mechanism for H_2O involved cycle.

Supplementary Note 1. DFT calculations

We performed density functional theory (DFT)[1] calculations in Gaussian 09[2] for key species using the B3LYP functional[3] and Lanl2dz basis set. The calculations were performed in vacuum to gain mechanistic insight. Transition states were found with the Synchronous Transit-Guided Quasi-Newton (STQN) method[4] implemented in Gaussian.

The key reaction step is proton transfer between the water oxygen and the methoxy group. In the presence of Br^- (Supplementary Table 7), upon formation of cation intermediate **III** (Supplementary Figure 8), there is Br^--assisted formation of the complex with water, which plays the role of the initial state to proton transfer to the methoxy with a barrier of 1.29/1.15 eV (internal/free energy) to form HBr and MeOH with barrierless separation of MeOH.

However, in the absence of Br^- (Supplementary Figure 9 and Table 8), complexation of the fullerene with water is not favored. In this case, a complex with HO^- (present due to partial dissociation of water) may be formed which can play the role of the initial state for proton transfer to the methoxy, with a higher barrier of 1.46/1.26 eV (internal/free energy). This pathway is thus less preferred kinetically, thermodynamically, and sterically (low concentration of HO^-).

Therefore, ketone synthesis with formation of MeOH and HBr is effectively accelerated by Br^-. The computed mechanism also confirms that the ketone oxygen directly comes from water rather than the methoxy group.

Supplementary Table 7 DFT computed results for intra-molecular proton transfer in the presence of Br^-.

Reaction state	Molecular model	Internal energy (eV)	Free energy (eV)[a]
Initial state	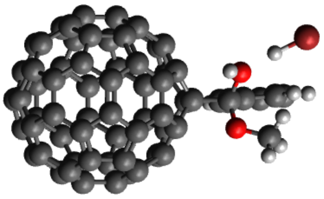	0[b]	0
Transition state	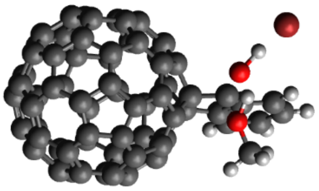	1.29	1.15
Product state	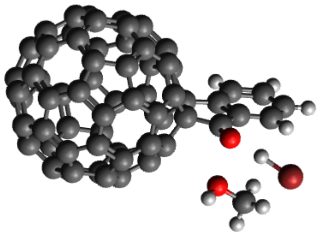	– 0.16	– 0.34

Atom color scheme here and elsewhere: C–dark grey, H–light grey, O–red, Br–magenta; [a]Free energies were computed at 300 K. [b]The energy of initial state was defined as 0 for reference.

Supplementary Table 8. DFT computed results for intra-molecular proton transfer in the absence of Br⁻.

Reaction state	Molecular model	Internal energy (eV)	Free energy (eV)[a]
Initial state	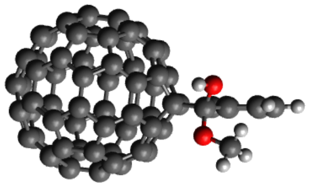	0[b]	0
Transistion state	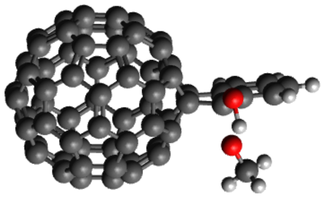	1.46	1.26
Product state	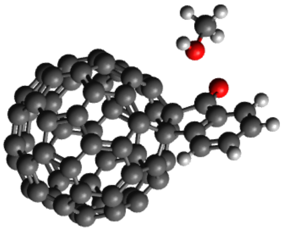	– 0.09	– 0.29

Atom color scheme here and elsewhere: C–dark grey, H–light grey, O–red, Br–magenta; [a]Free energies were computed at 300 K. [b]The energy of initial state was defined as 0 for reference.

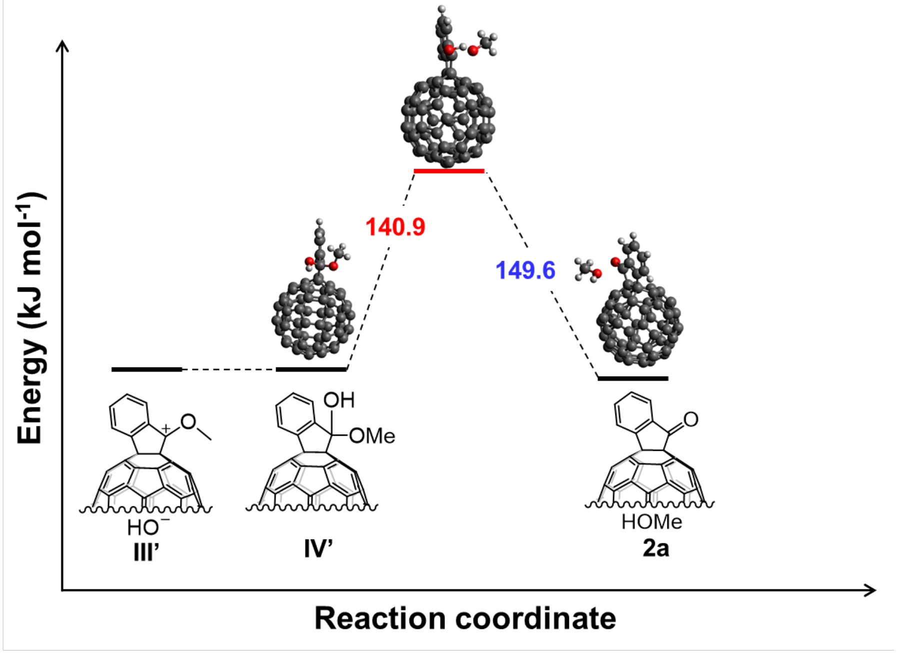

Supplementary Figure 8. DFT computed energy change from III to 2a in the absence of Br⁻.

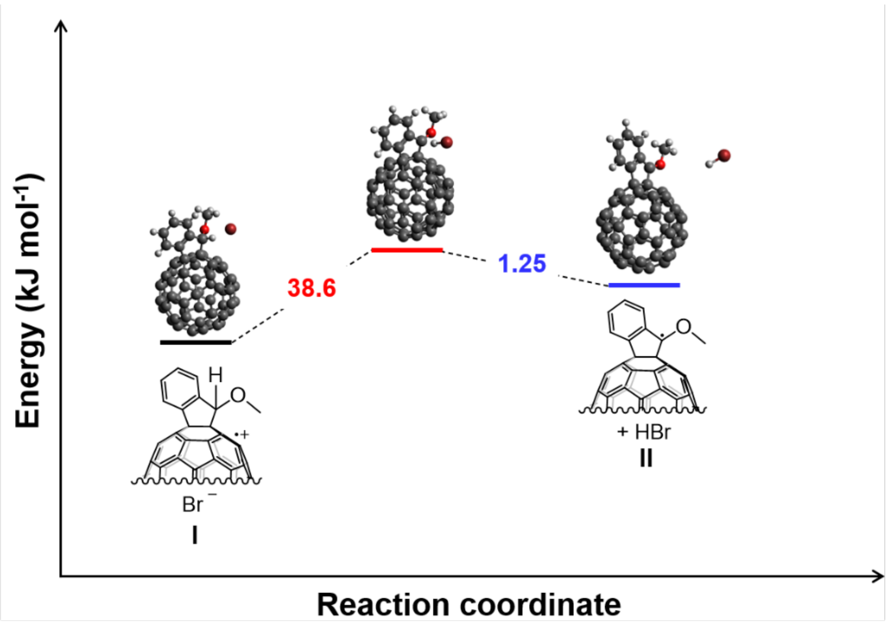

Supplementary Figure 9. DFT computed energy change from I to II in the presence of Br⁻.

The calculations also show oxidation of **1a** in the presence of Cu[II], as can be seen from the Mulliken charges on Cu and on **1a** when Cu(II) coordinates to **1a** are about + 0.7 for Cu and + 1.3 for the fullerene. The proton transfer barrier from **I** (Supplementary Figure 8) to Br⁻ to form HBr is computed to be only 0.40/0.32 eV (internal/free energy), confirming that the rate-determine step is proton transfer occurred in step from **III** to **2a**, rather than the step from **I** to **II** (Supplementary Fig. 9).

4. Evaporable fullerene-fused ketone

HPLC analyses

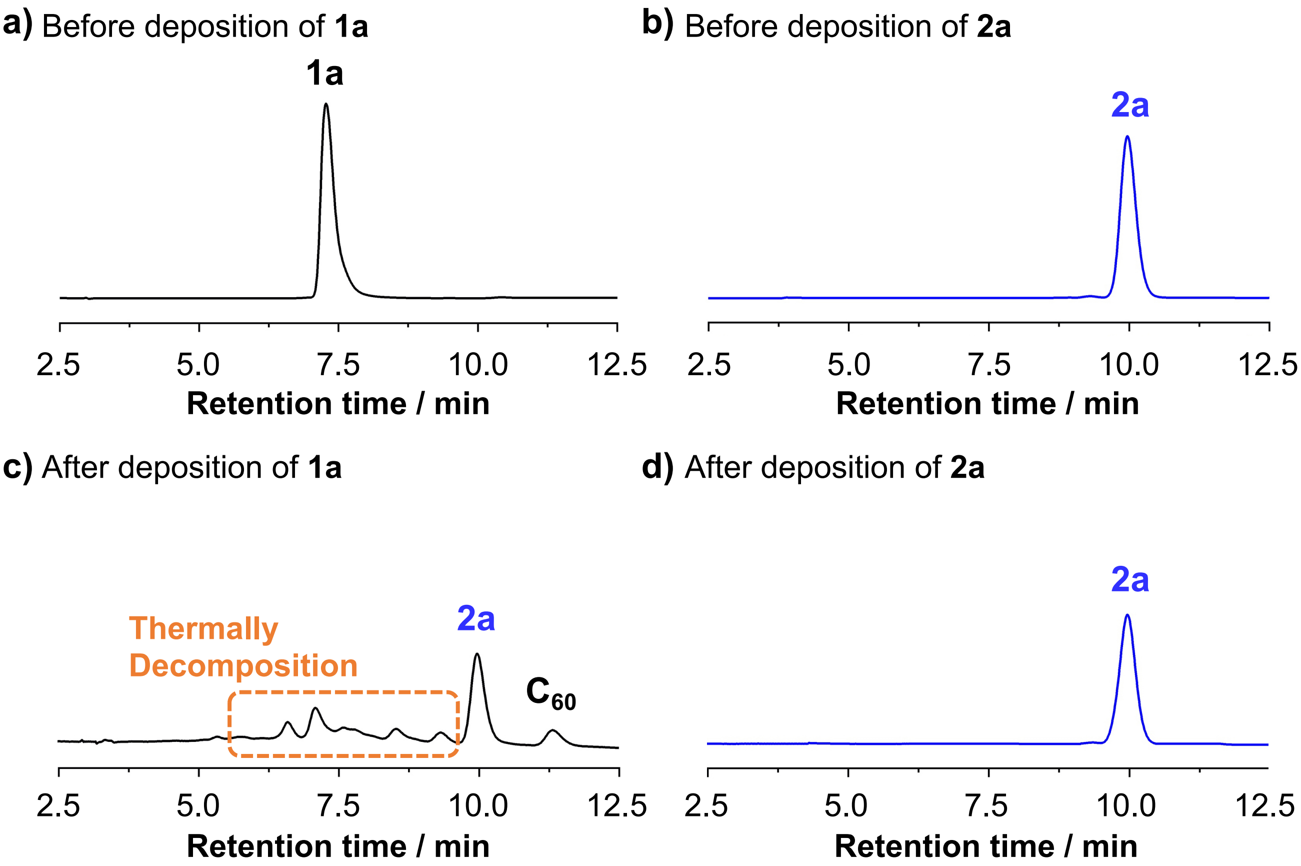

Supplementary Figure 10. HPLC analyses. **a)** Before deposition of **1a**, **b)** Before deposition of **2a**, **c)** After deposition of **1a**, **d)** After deposition of **2a**.

TGA analyses

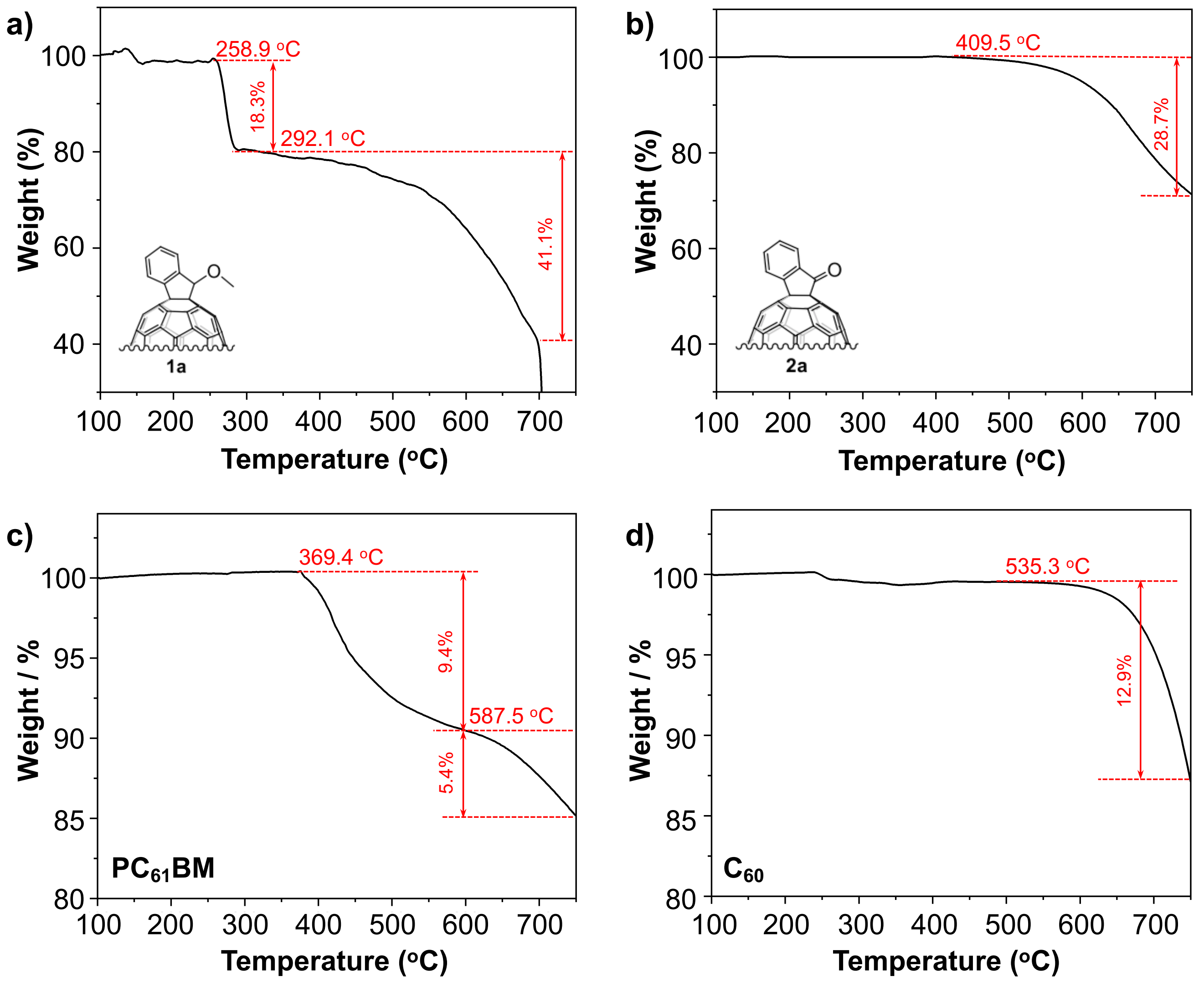

Supplementary Figure 11. TGA measurements. Under a N_2 gas flow with temperature ramp rate of 10 °C/min until 800 °C: **a)** indano[60]fullerene **1a**, **b)** [60]fullerene-fused ketone **2a**, **c)** $PC_{61}BM$, **d)** C_{60}.

TEM and SAED observations

TEM sample preparation. Spin-coated **2a**-film was prepared by spin-coating saturated **2a** *ortho*-dichlorobenzene with a 0.22 µm filter to remove any undissolved particles. After spin-coating onto the TEM grid, the grid was annealed at 100 °C to remove excess solvent. Vacuum-deposited **2a**-film was prepared directly from depositing **2a** by a thermal evaporator.

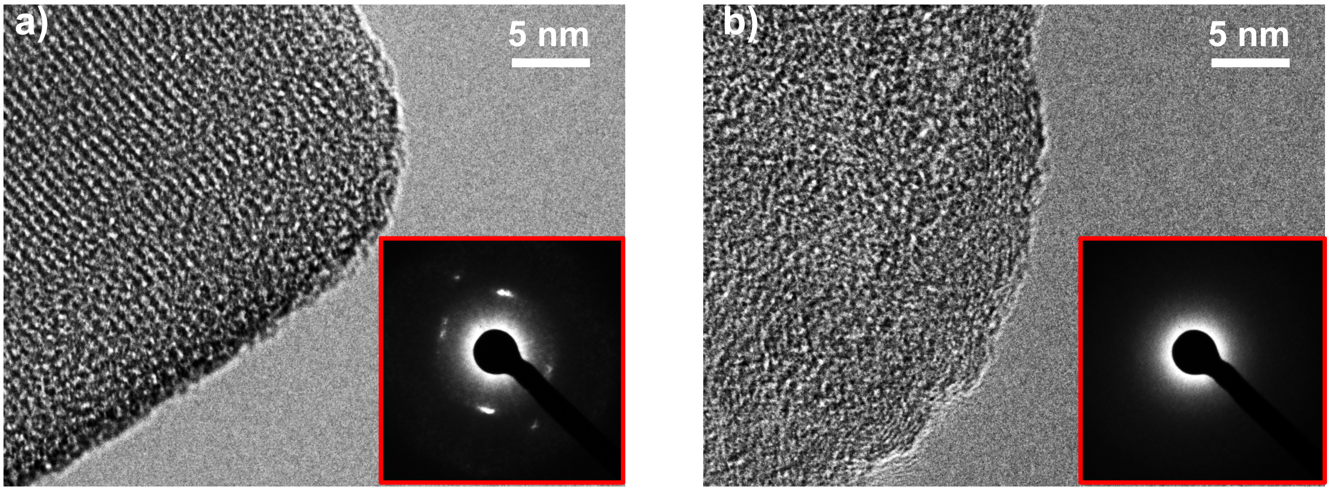

Supplementary Figure 12. TEM observations. **a)** spin-coated **2a**-film and **b)** vacuum-deposited **2a**-film with the selected area electron diffraction (SAED) as an inset.

UV-Vis observations

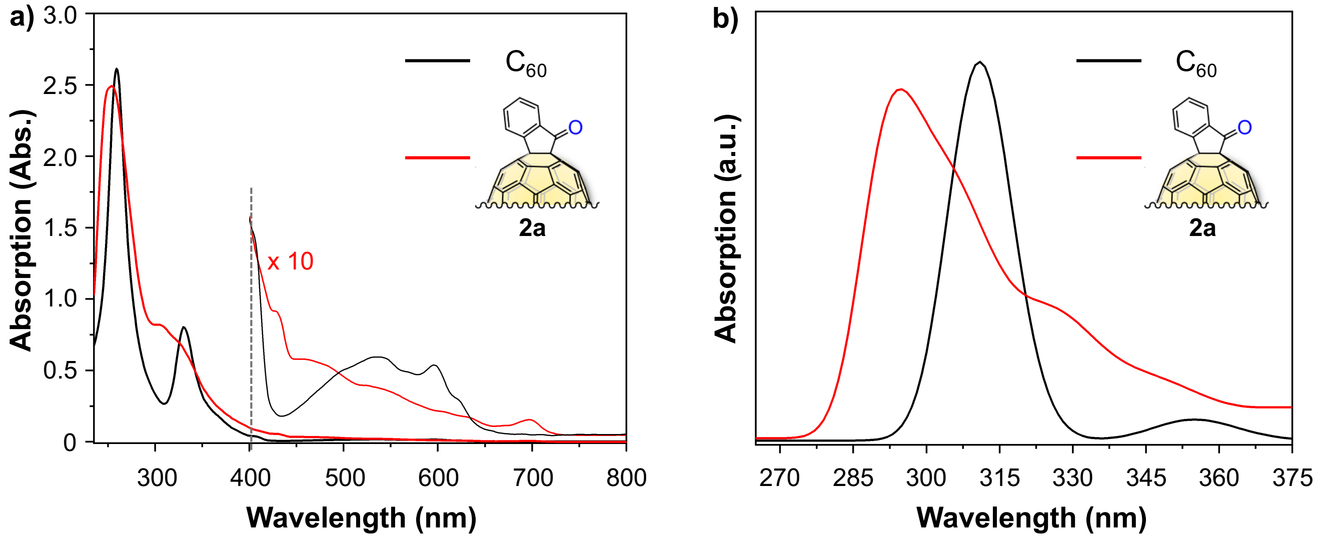

Supplementary Figure 13. UV-Vis spectra. a) C_{60}-film (black) and **2a**-film (red) through vacuum-deposition by 30 nm on bare glass substrate. **b)** DFT computational UV-vis of C_{60} (black) and **2a** (red).

Energy levels of evaporable fullerene-fused ketones 2a–d

Supplementary Table 9 Half-wave reduction potentials and LUMO levels of fullerenes.[a]

Fullerenes	$E_{1/2}^{red}$ (V vs Fc/Fc$^+$)		LUMO level (eV)[b]
	E_1	E_2	
C_{60}	–1.11	–1.50	–3.69
**2a**	–1.13	–1.51	–3.67
**2b**	–1.14	–1.51	–3.66
**2c**	–1.12	–1.50	–3.68
**2d**	–1.15	–1.52	–3.65

[a]Potentials in eV vs a ferrocene/ferrocenium (Fc/Fc$^+$) couple were recorded by cyclic voltammetry in *o*-DCB solution containing $Bu_4N^+(CF_3SO_2)_2N^-$ (0.1 M) as supporting electrolyte at 25 °C with a scan rate of 0.05 V/s. Platinum disk, platinum wire, and Ag/Ag$^+$ electrodes were used as the working, counter, and reference electrodes, respectively. [b]Estimated LUMO levels using the following equation: LUMO level = – (4.8 + E_1) eV.[6]

Supplementary Table 10 Comparison of half-wave reduction potentials and LUMO levels for 1a–d and 2a–d.

1[a]	E_1 (V)	LUMO level (eV)	2	E_1 (V)	LUMO level (eV)
**1a**	–1.20	–3.60	**2a**	–1.13	–3.67
**1b**	–1.21	–3.59	**2b**	–1.14	–3.66
**1c**	–1.19	–3.61	**2c**	–1.12	–3.68
**1d**	–1.23	–3.57	**2d**	–1.15	–3.65

[a]Half-wave potentials and LUMO levels of **1a–d** are cited from our previous research.[7]

Supplementary Table 11 Computed HOMO and LUMO levels of 2a–d.

Fullerenes	HOMO orbitals	HOMO levels (eV)	LUMO orbitals	LUMO levels (eV)
C_{60}	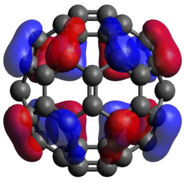	–6.723	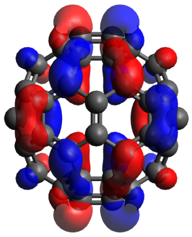	–3.893
$PC_{61}BM$	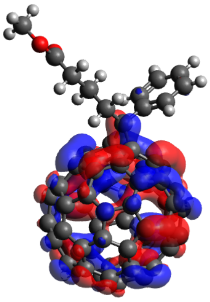	–6.319	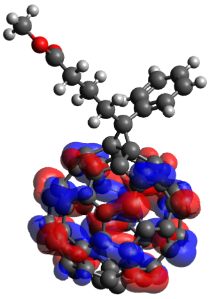	–3.700
**2a**	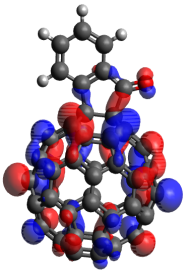	–6.392	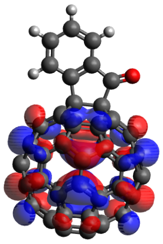	–3.805
**2b**	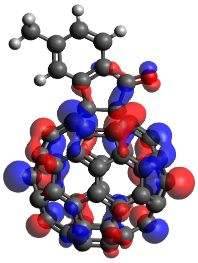	–6.356	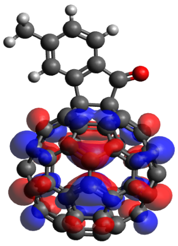	–3.773
**2c**	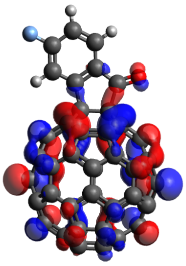	–6.483	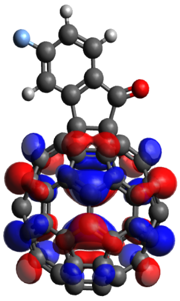	–3.885
**2d**	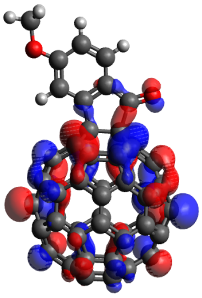	–6.316	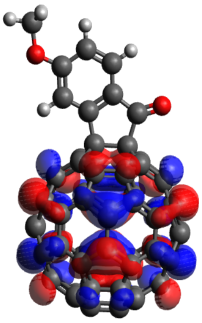	–3.733

5. Supplementary Methods

General methods

Unless otherwise noted, all materials including dry solvents were obtained from commercial suppliers (Sigma-Aldric, TCI, Wako) and used without further purification. Unless otherwise noted, all reactions were performed with dry solvents under an atmosphere of argon in flame-dried glassware with standard vacuum-line techniques or in a glove box. The reaction monitoring was conducted by an analytical high-performance liquid chromatography (HPLC), using a COSMOSIL Buckyprep-D column as solid state, toluene or toluene/isopropanol as eluent with a flow rate of 1.0 mL/min under the detection wavelength at 337 nm.

All NMR spectra were taken at room temperature by 400 MHz (Bruker AVANCE III 400 spectrometer), 500 MHz (Bruker AVANCE III 500 spectrometer) or 600 MHz (Bruker AVANCE III 600 spectrometer). Unless otherwise specified, all the NMR spectra were recorded in parts per million (ppm, scale) with the proton of $CDCl_3$ (7.260 ppm) or for ^1H NMR and carbon of $CDCl_3$ (77.16 ppm) for ^{13}C NMR as internal reference, respectively. The data were presented as following order: chemical shift, multiplicity (s = singlet, d = doublet, t = triplet, hept = heptet, m = multiplet and/or multiplet resonances), coupling constant in hertz (Hz), and signal area integration in natural numbers, assignment (*italic*). High-resolution mass spectra (HRMS) were obtained by MALDI using a time-of-flight mass analyzer on a Bruker Ultra exTOF/TOF spectrometer.

Spectra data of products 2a–d

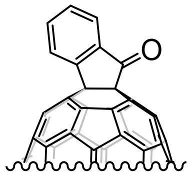

2a

Spectra data of 2a. Column chromatography with CS_2 as eluent afforded product **2a** (23.2 mg, 94%) as black crystal powder. ^1H NMR (400 MHz, $CS_2/CDCl_3$) δ 8.534 (dd, J_1 = 8.0 Hz, J_2 = 0.4 Hz, 1H), 8.456 (dd, J_1 = 7.6 Hz, J_2 = 0.4 Hz, 1H), 8.102 (dt, J_1 = 7.6 Hz, J_2 = 1.2 Hz ,1H), 7.892 (dt, J_1 = 8.0 Hz, J_2 = 1.2 Hz, 1H); $^{13}C\{^1H\}$ NMR (150 MHz, $CS_2/CDCl_3$, all 2C unless indicated) δ 181.31 (C=O, 1C), 140.47 (*aryl C*, 1C), 138.47, 138.16, 133.57, 133.37 (1C), 133.15 (1C), 132.53, 132.52, 132.34, 132.29, 132.09, 131.94, 131.88, 131.83, 131.75, 131.73, 131.14, 130.85 (1C), 129.94, 129.65, 129.60, 129.37, 129.15, 129.11, 129.10 (*aryl C*, 1C), 128.95, 128.79, 127.95, 127.88, 125.04 (*aryl C*, 1C), 123.86 (1C), 123.78 (1C), 122.86 (1C), 119.60

(*aryl C*, 1C), 117.35(*aryl C*, 1C), 116.85 (*aryl C*, 1C), 79.47 (sp^3-*C* of C_{60}, 1C), 77.33 (sp^3-*C* of C_{60}, 1C); MALDI-TOF MS *m/z* calcd for $C_{67}H_4O$ $[M]^+$ 824.0262 , found 824.0260.

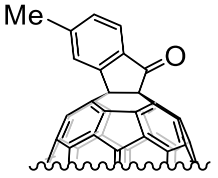

2b

Spectra data of 2b. Column chromatography with CS_2/DCM (4/1) as eluent afforded product **2b** (23.4 mg, 93%) as black crystal powder. ^{1}H NMR (500 MHz, $CS_2/CDCl_3$) δ 8.331 (d, J = 8.0 Hz, 1H), 8.323 (s, 1H), 7.684 (dd, J_1 = 7.5 Hz, J_2 = 1.0 Hz, 1H), 2.758 (s, 3H); MALDI-TOF MS *m/z* calcd for $C_{68}H_6O$ $[M]^+$ 838.0419, found 838.0413, which is in accordance with the reported literature.[8]

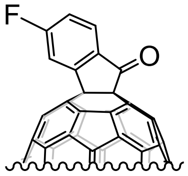

2c

Spectra data of 2c. Column chromatography with CS_2/DCM (2/1) as eluent afforded product **2c** (23.2 mg, 92%) as black crystal powder. ^{1}H NMR (500 MHz, $CS_2/CDCl_3$) δ 8.484 (dd, J_1 = 8.5 Hz, J_2 = 5.5 Hz ,1H), 8.180 (dd, J_1 = 8.0 Hz, J_2 = 2.0 Hz ,1H), 7.582 (dt, J_1 = 8.5 Hz, J_2 = 2.0 Hz ,1H); ^{13}C NMR (150 MHz, $CS_2/CDCl_3$, all 2C unless indicated) δ 199.41 (C=O, 1C), 168.54 (*aryl C*, J = 260.6 Hz, 1C), 159.27 (1C), 153.21, 152.59, 147.48, 147.41, 147.13, 146.37, 146.34, 146.11, 146.05, 145.62 (3C), 145.54, 145.52, 145.48, 145.39, 145.34, 144.58, 144.20, 143.10 (3C), 142.75, 142.69, 142.38, 142.25 (1C), 142.07, 142.04, 141.98, 141.87, 141.64, 140.63 (*aryl C*, J = 6.0 Hz, 1C), 135.48 (*aryl C*, J = 43.6 Hz, 1C), 134.42 (1C), 130.33 (1C), 129.63 (*aryl C*, J = 10.4 Hz, 1C), 118.78 (*aryl C*, J = 23.7 Hz, 1C), 113.70 (*aryl C*, J = 23.1 Hz, 1C), 73.87 (sp^3-*C* of C_{60}, 1C), 71.58 (sp^3-*C* of C_{60}, 1C); MALDI-TOF MS *m/z* calcd for $C_{67}H_3OF$ $[M]^+$ 842.0168, found 842.0165.

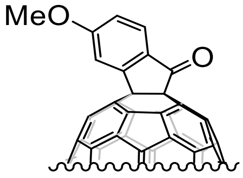

2d

Spectra data of 2d. Column chromatography with CS_2/DCM (2/1) as eluent afforded product **2d** (24.1 mg, 94%) as black crystal powder. ^1H NMR (500 MHz, CS_2/$CDCl_3$) δ 8.382 (d, J = 8.5 Hz,1H), 7.825 (d, J = 2.5 Hz ,1H), 7.650 (dd, J_1 = 8.5 Hz, J_2 = 2.5 Hz ,1H), 4.124 (s, 3H); MALDI-TOF MS *m/z* calcd for $C_{68}H_6O_2$ $[M]^+$ 854.0368, found 854.0372, which is in accordance with the reported literature.[8]

6. NMR spectra

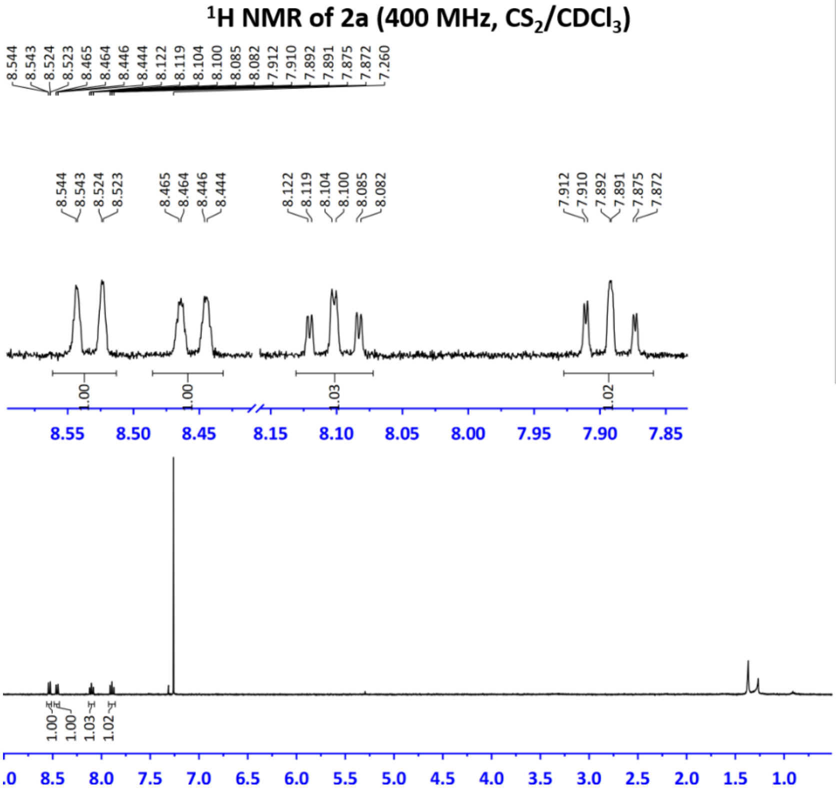

	Parameter	Value
1	Comment	
2	Origin	Bruker BioSpin GmbH
3	Owner	nmrsu
4	Instrument	spect
5	Solvent	CDCl3
6	Temperature	293.2
7	Pulse Sequence	zg30
8	Experiment	1D
9	Number of Scans	8
10	Receiver Gain	166.2
11	Relaxation Delay	1.0000
12	Pulse Width	10.0000
13	Presaturation Frequency	
14	Acquisition Time	4.0894
15	Acquisition Date	2019-02-23T18:51:00
16	Modification Date	2019-02-23T18:51:06
17	Spectrometer Frequency	400.03
18	Spectral Width	8012.8
19	Lowest Frequency	-1813.9
20	Nucleus	1H
21	Acquired Size	32768
22	Spectral Size	65536

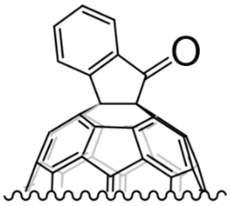

2a

Supplementary Figure 14. ^{1}H NMR of 2a.

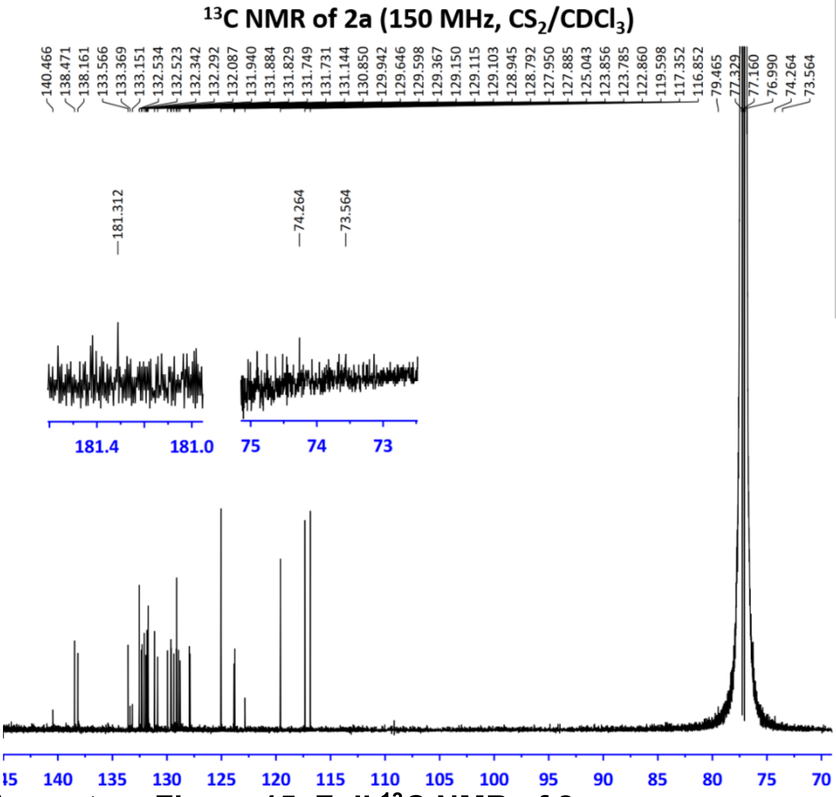

	Parameter	Value
1	Instrument	JNM-ECA600
2	Author	delta
3	Solvent	CHLOROFORM-D
4	Temperature	22.7
5	Pulse Sequence	carbon.jxp
6	Probe	2756
7	Number of Scans	21009
8	Receiver Gain	56.0
9	Relaxation Delay	2.0000
10	Pulse Width	3.4333
11	Acquisition Time	0.6921
12	Acquisition Date	2017-10-31T19:46:01
13	Modification Date	2017-11-01T11:28:54
14	Spectrometer Frequency	150.91
15	Spectral Width	37878.8
16	Lowest Frequency	-3876.3
17	Nucleus	13C
18	Acquired Size	32768
19	Spectral Size	52430

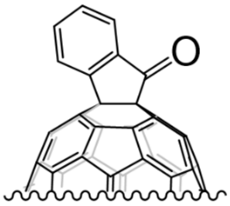

2a

Supplementary Figure 15. Full ^{13}C NMR of 2a.

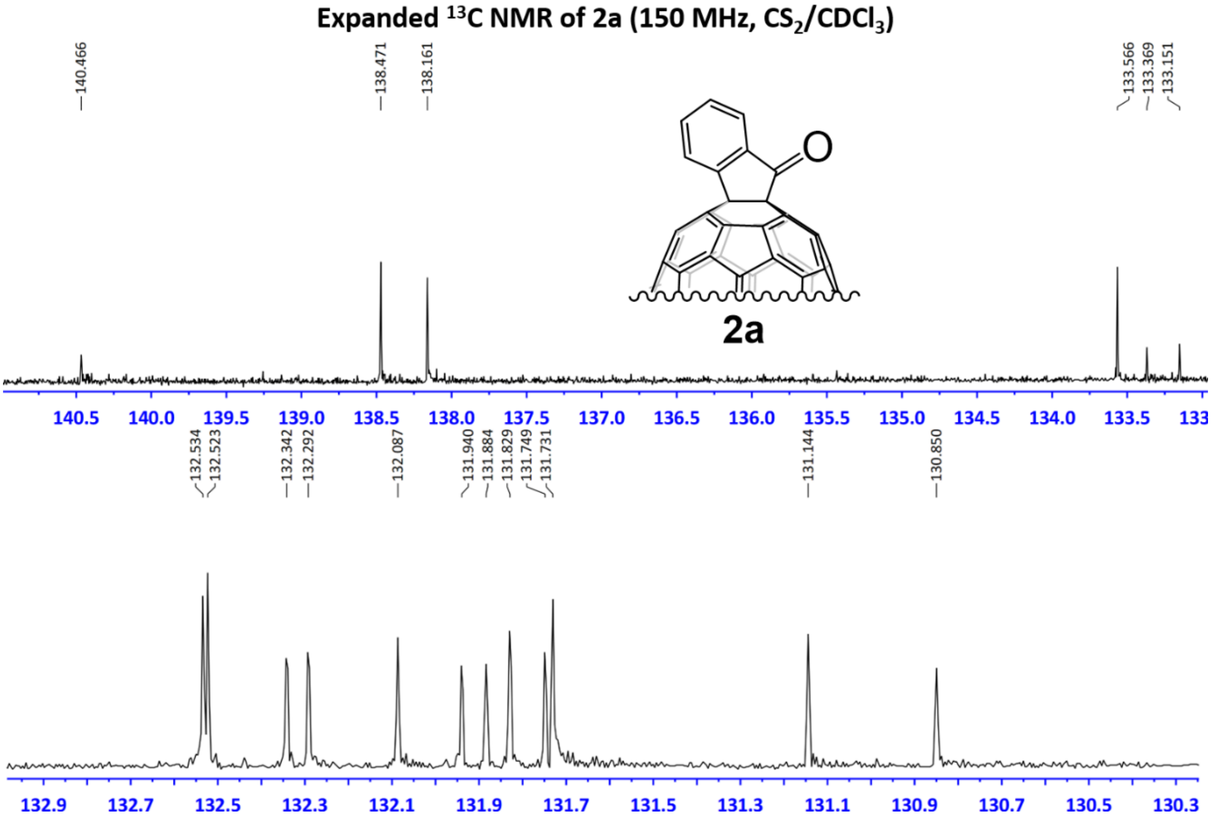

Supplementary Figure 16. Expanded ^{13}C NMR of 2a at a range of 140–130 ppm.

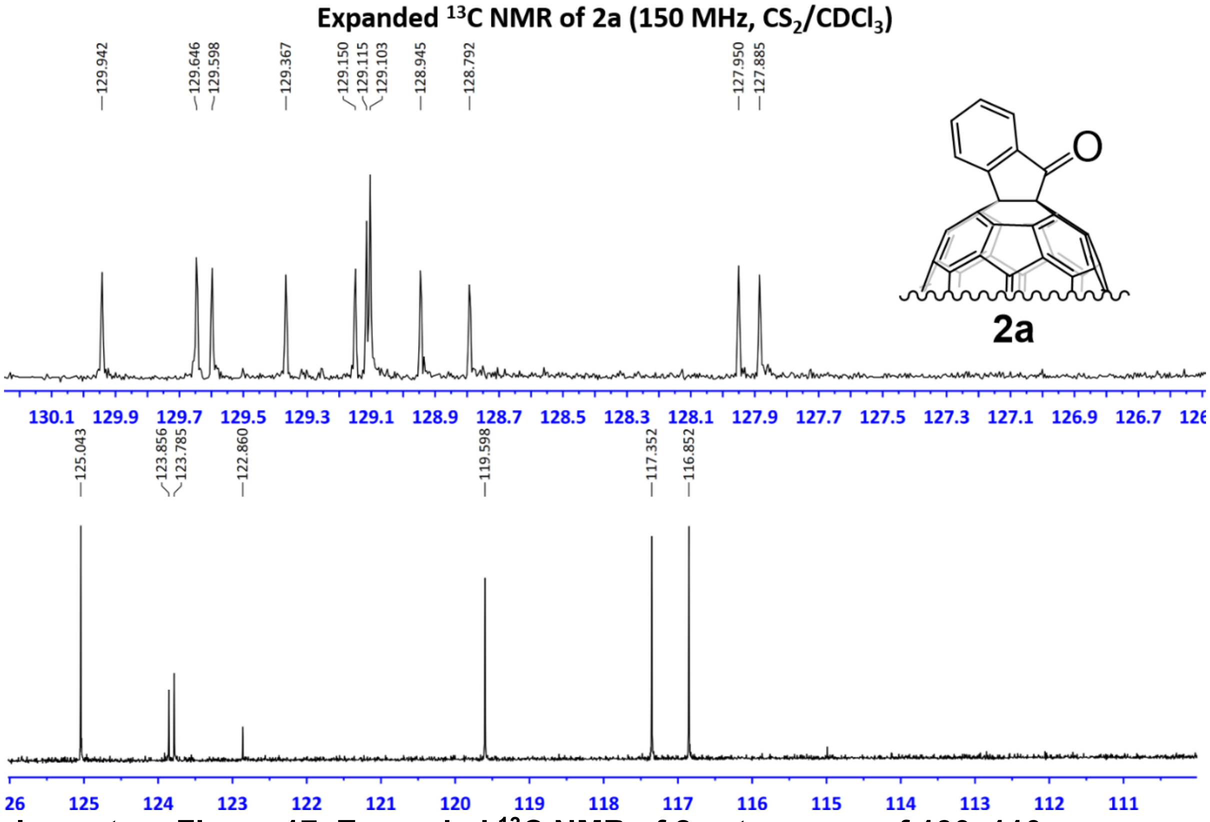

Supplementary Figure 17. Expanded ^{13}C NMR of 2a at a range of 130–110 ppm.

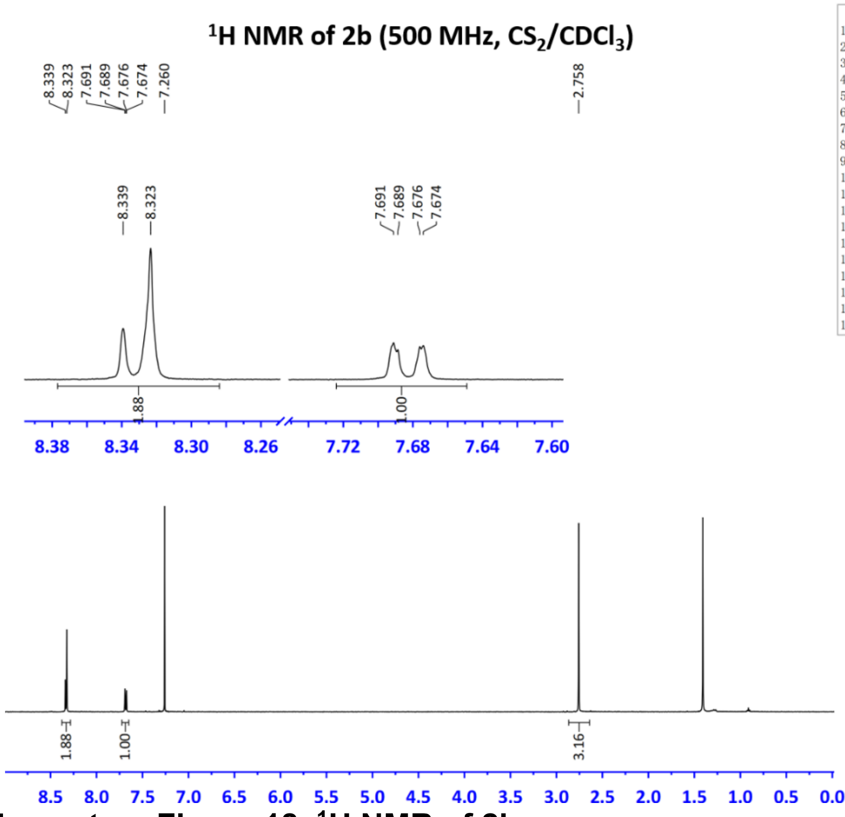

	Parameter	Value
1	Instrument	JNM-ECZ500R/ S1
2	Author	delta
3	Solvent	CHLOROFORM-D
4	Temperature	19.3
5	Pulse Sequence	single_pulse.jxp
6	Probe	2774
7	Number of Scans	16
8	Receiver Gain	50.0
9	Relaxation Delay	5.0000
10	Pulse Width	3.1500
11	Acquisition Time	1.7459
12	Acquisition Date	2019-06-04T19:04:15
13	Modification Date	2019-06-04T19:06:12
14	Spectrometer Frequency	500.16
15	Spectral Width	7507.5
16	Lowest Frequency	-1247.5
17	Nucleus	1H
18	Acquired Size	16384
19	Spectral Size	52430

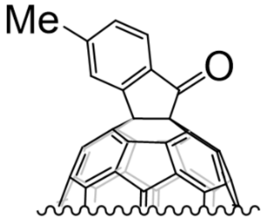

2b

Supplementary Figure 18. ^{1}H NMR of 2b.

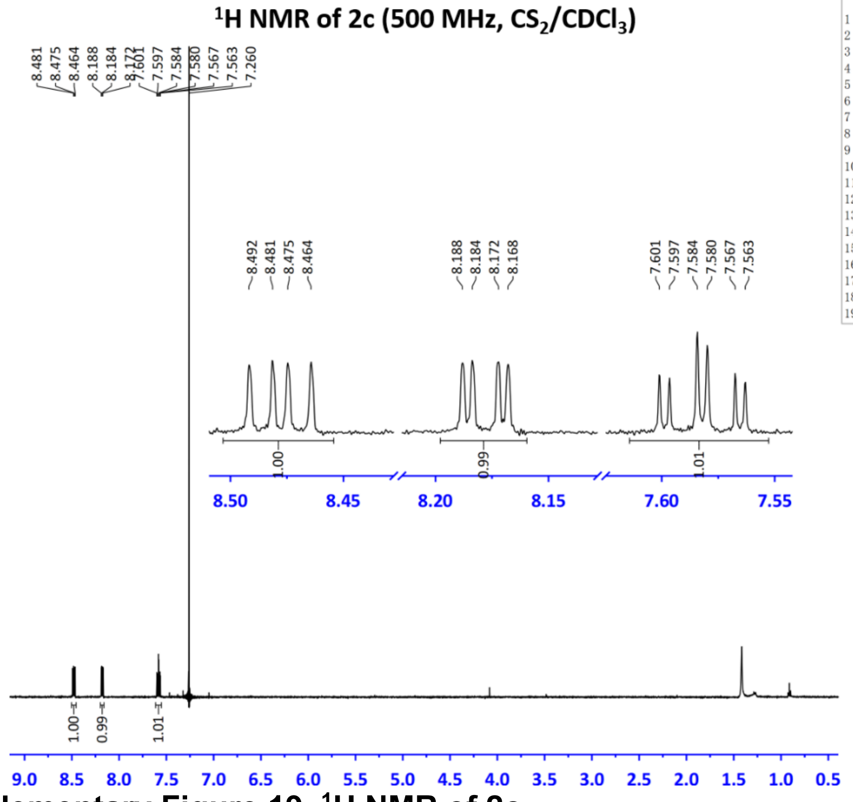

	Parameter	Value
1	Instrument	JNM-ECZ500R/ S1
2	Author	delta
3	Solvent	CHLOROFORM-D
4	Temperature	18.7
5	Pulse Sequence	single_pulse.jxp
6	Probe	2774
7	Number of Scans	8
8	Receiver Gain	76.0
9	Relaxation Delay	5.0000
10	Pulse Width	3.1500
11	Acquisition Time	1.7406
12	Acquisition Date	2019-05-10T14:36:37
13	Modification Date	2019-05-10T14:37:41
14	Spectrometer Frequency	500.16
15	Spectral Width	7530.1
16	Lowest Frequency	-1258.8
17	Nucleus	1H
18	Acquired Size	16384
19	Spectral Size	52430

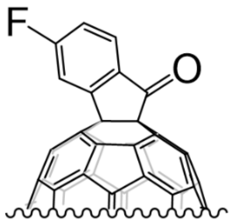

2c

Supplementary Figure 19. ^{1}H NMR of 2c.

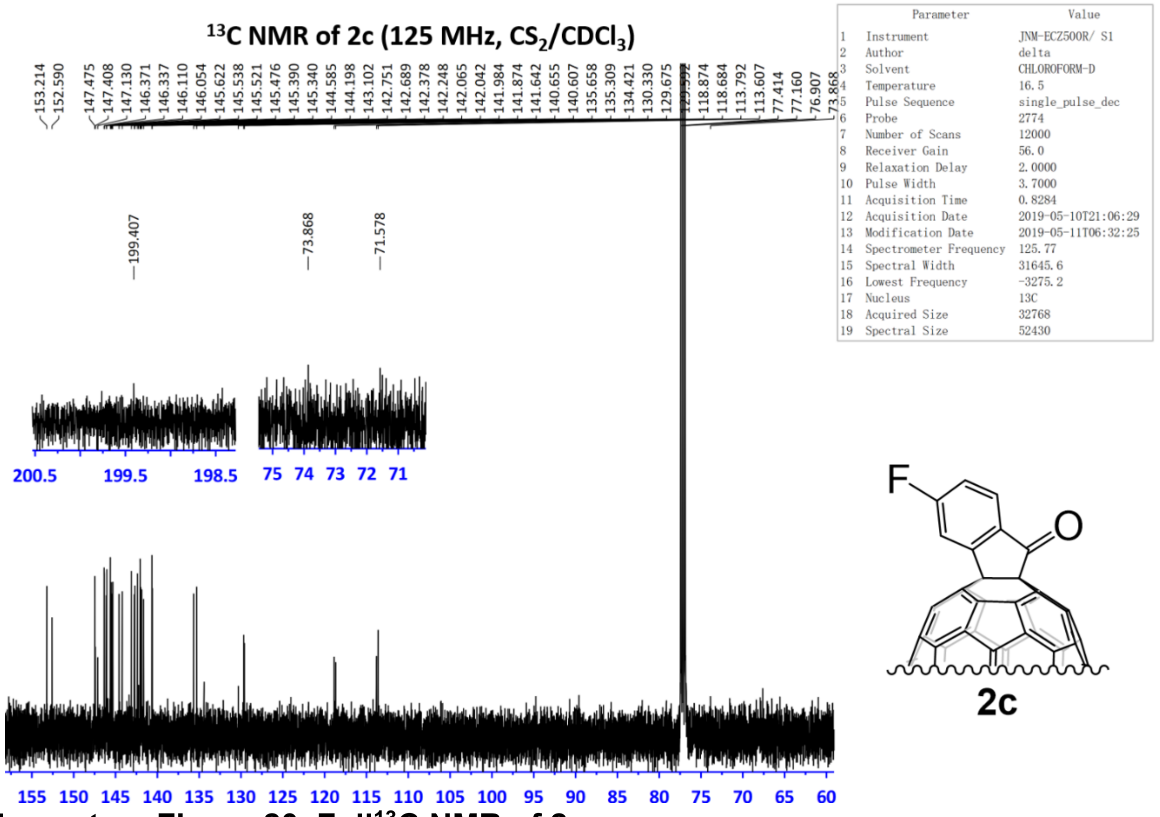

Supplementary Figure 20. Full^{13}C NMR of 2c.

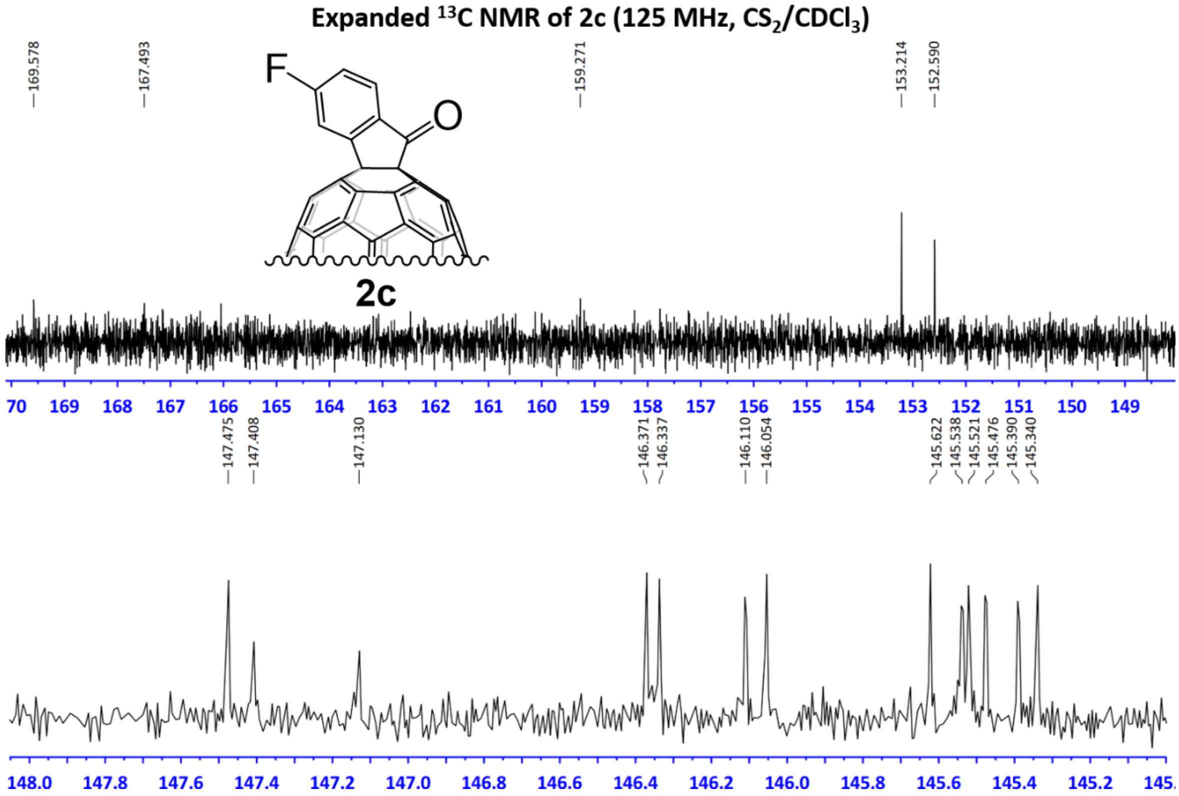

Supplementary Figure 21. Expanded ^{13}C NMR of 2c at a range of 170–145 ppm.

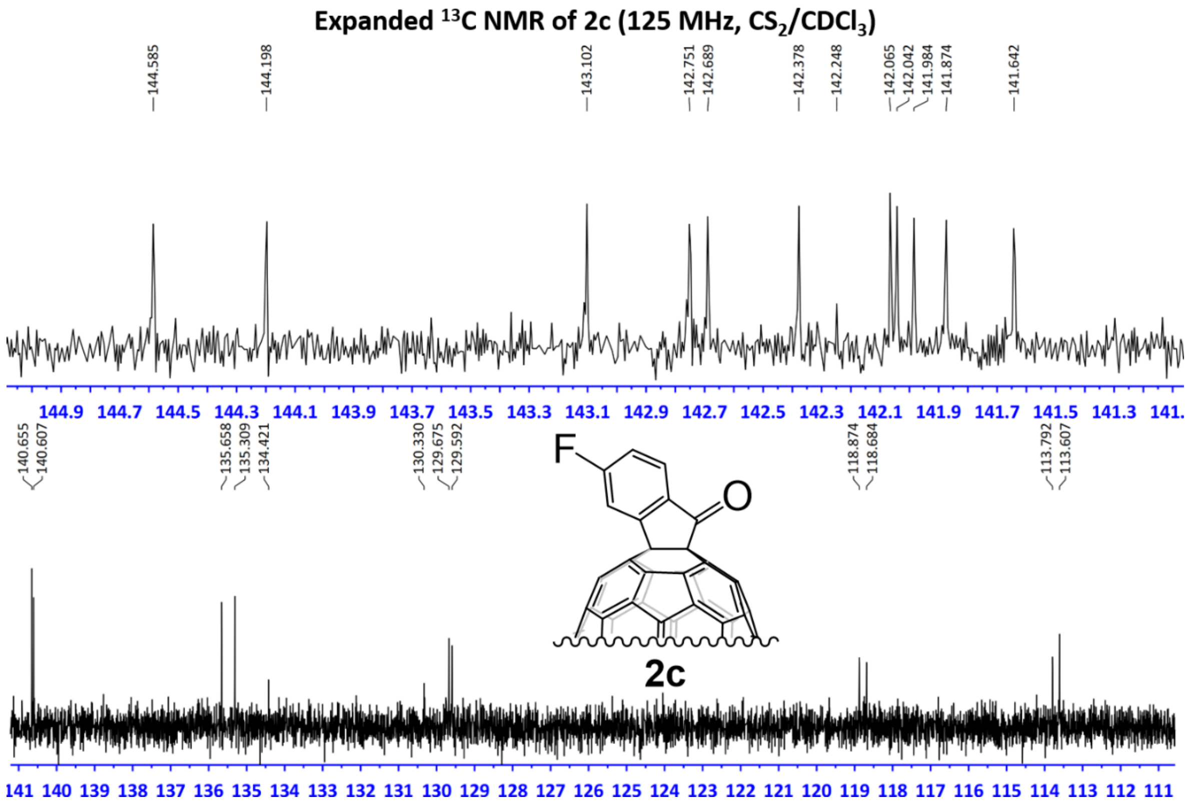

Supplementary Figure 22. Expanded ^{13}C NMR of 2c at a range of 145–110 ppm.

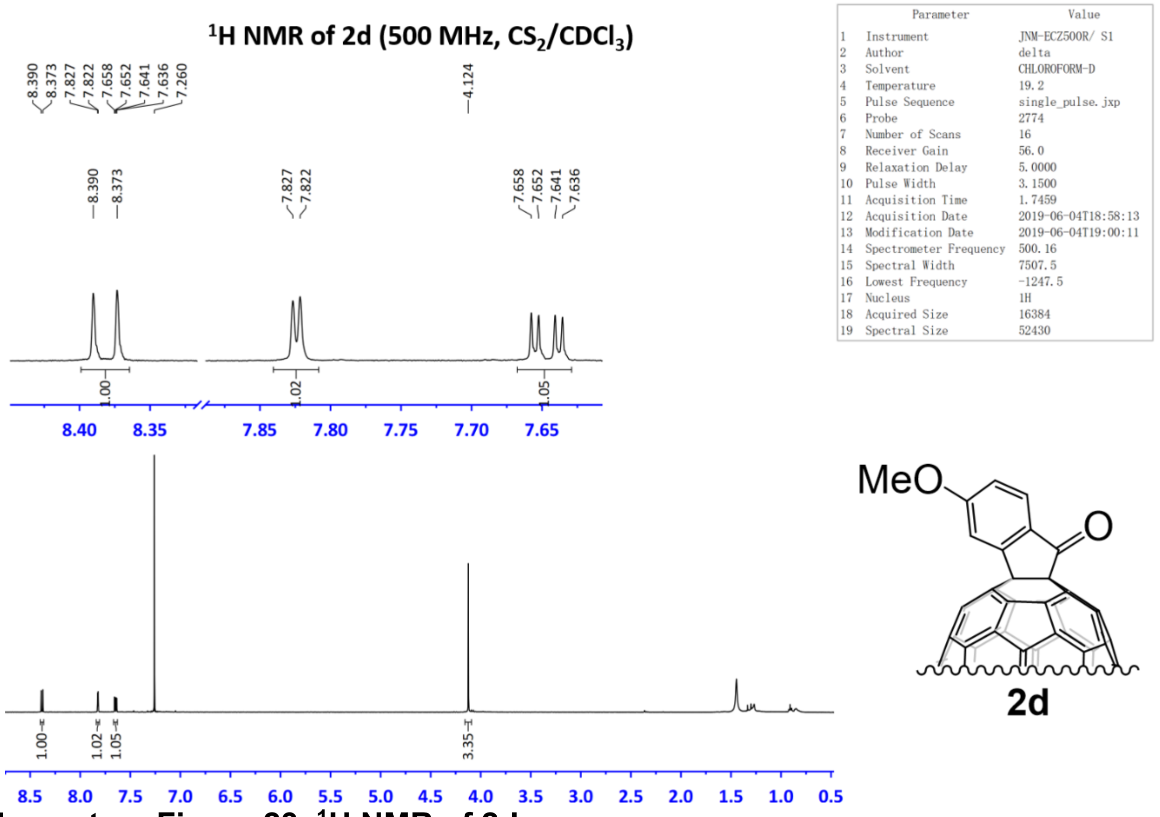

Supplementary Figure 23. ^{1}H NMR of 2d.

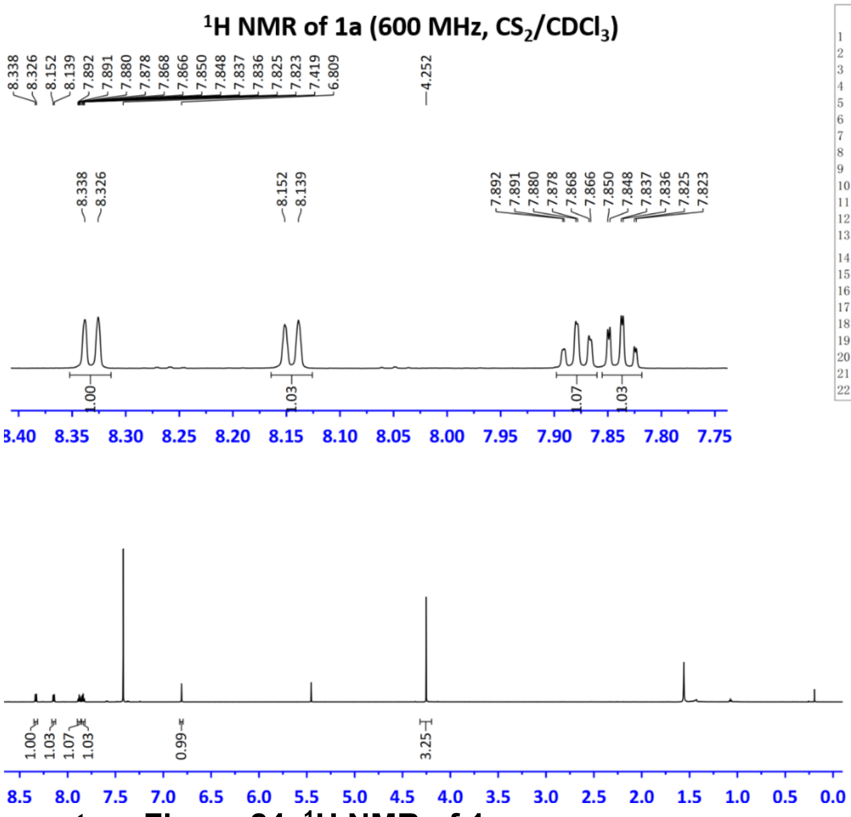

Parameter	Value
1 Comment	D7954 H1
2 Origin	Bruker BioSpin GmbH
3 Owner	nmrsu
4 Instrument	spect
5 Solvent	CDCl3
6 Temperature	299.0
7 Pulse Sequence	zg30
8 Experiment	1D
9 Number of Scans	64
10 Receiver Gain	171.3
11 Relaxation Delay	1.0000
12 Pulse Width	10.0000
13 Presaturation Frequency	
14 Acquisition Time	2.7263
15 Acquisition Date	2017-07-24T18:01:13
16 Modification Date	2017-07-24T18:01:13
17 Spectrometer Frequency	600.38
18 Spectral Width	12019.2
19 Lowest Frequency	-2340.2
20 Nucleus	1H
21 Acquired Size	32768
22 Spectral Size	65536

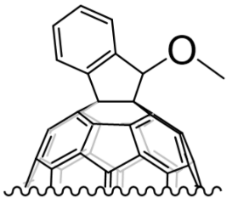

1a

Supplementary Figure 24. ^{1}H NMR of 1a.

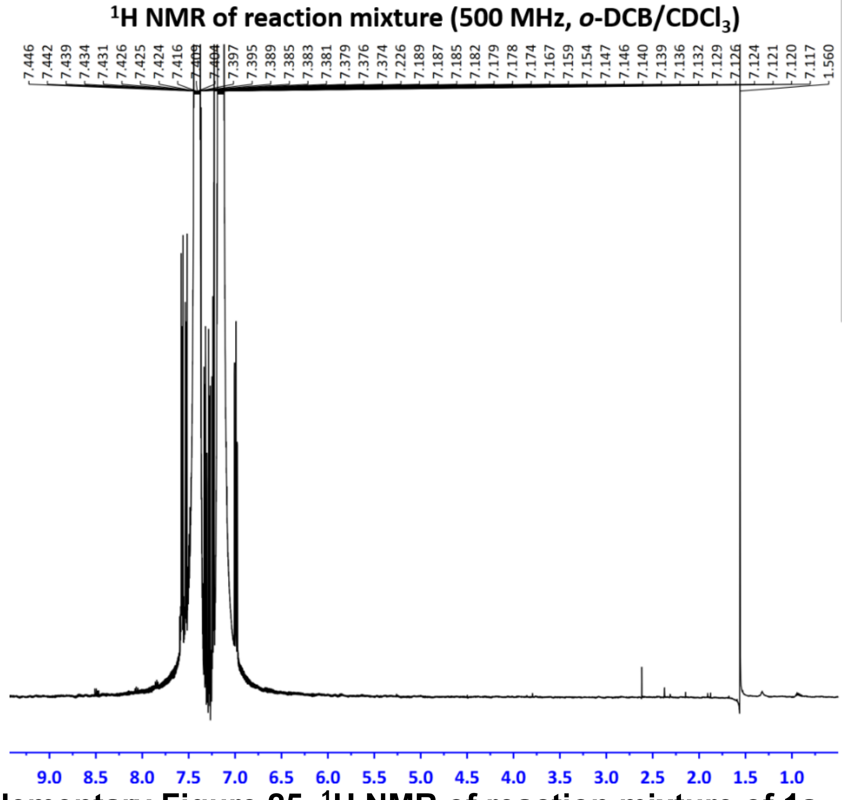

Parameter	Value
1 Instrument	JNM-ECZ500R/ S1
2 Author	delta
3 Solvent	CHLOROFORM-D
4 Temperature	17.6
5 Pulse Sequence	proton.jxp
6 Probe	2774
7 Number of Scans	64
8 Receiver Gain	26.0
9 Relaxation Delay	5.0000
10 Pulse Width	3.1500
11 Acquisition Time	1.7459
12 Acquisition Date	2020-03-04T12:04:10
13 Modification Date	2020-03-04T12:11:31
14 Spectrometer Frequency	500.16
15 Spectral Width	7507.5
16 Lowest Frequency	-1259.5
17 Nucleus	1H
18 Acquired Size	16384
19 Spectral Size	52430

Supplementary Figure 25. ^{1}H NMR of reaction mixture of 1a.

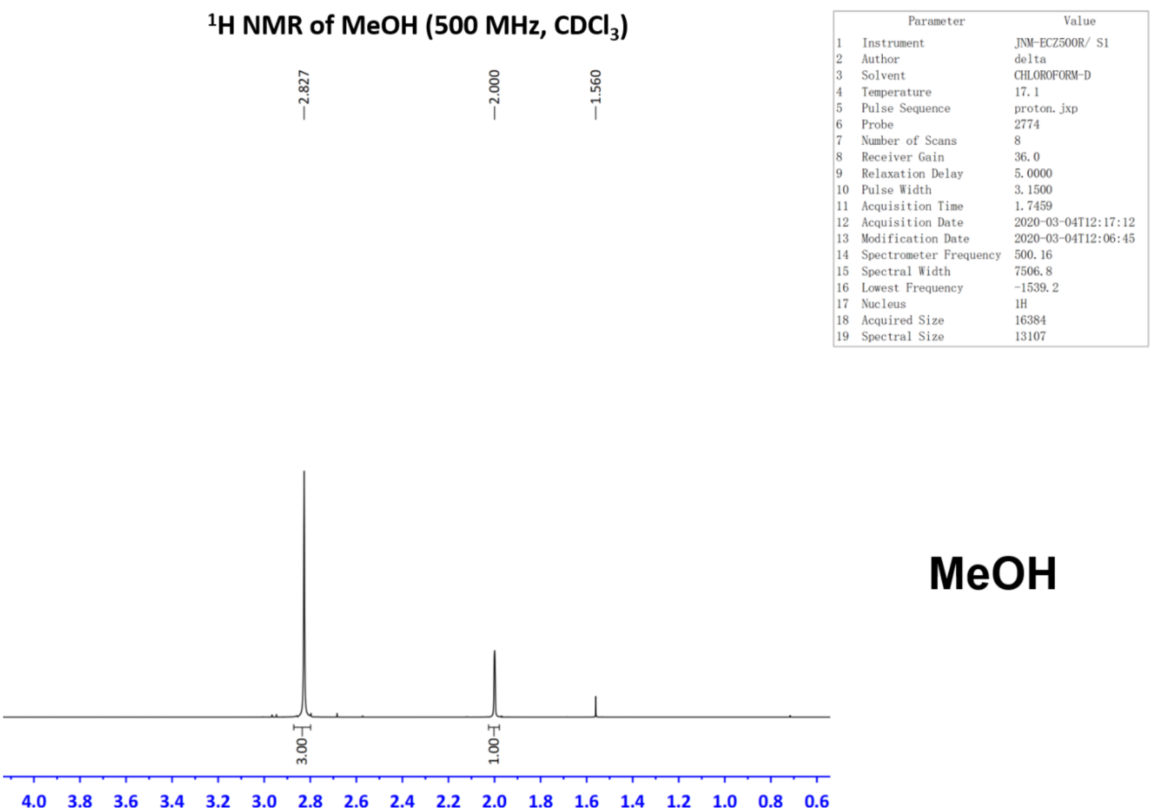

	Parameter	Value
1	Instrument	JNM-ECZ500R/ S1
2	Author	delta
3	Solvent	CHLOROFORM-D
4	Temperature	17.1
5	Pulse Sequence	proton.jxp
6	Probe	2774
7	Number of Scans	8
8	Receiver Gain	36.0
9	Relaxation Delay	5.0000
10	Pulse Width	3.1500
11	Acquisition Time	1.7459
12	Acquisition Date	2020-03-04T12:17:12
13	Modification Date	2020-03-04T12:06:45
14	Spectrometer Frequency	500.16
15	Spectral Width	7506.8
16	Lowest Frequency	-1539.2
17	Nucleus	1H
18	Acquired Size	16384
19	Spectral Size	13107

Supplementary Figure 26. ^{1}H NMR of MeOH.

7. HRMS spectra

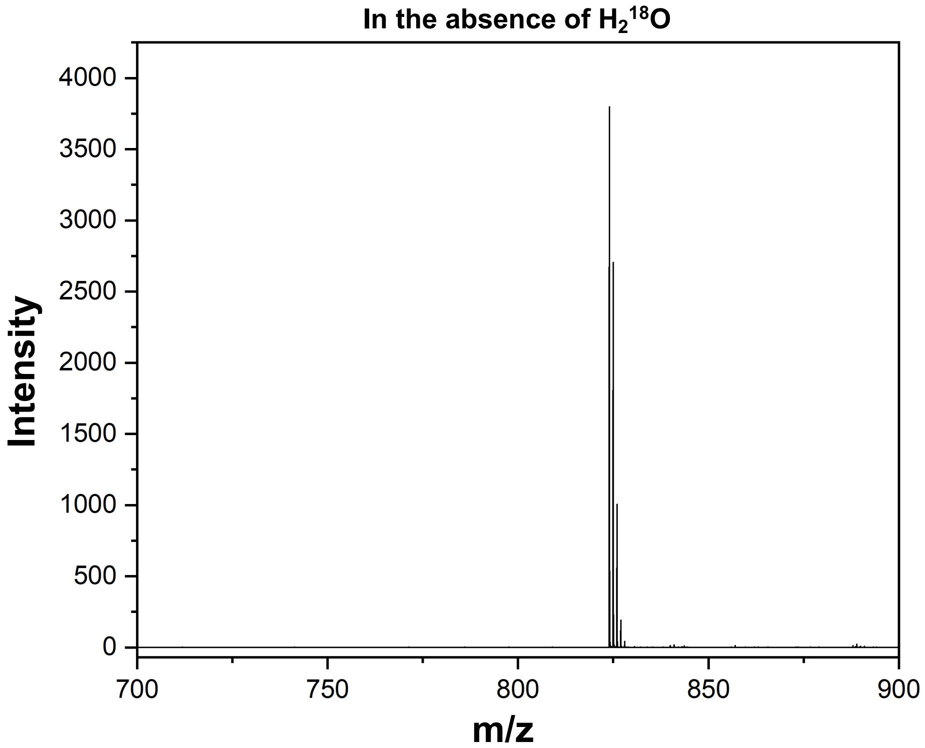

Supplementary Figure 27. Full HRMS of the reaction of 1a in the absence of $H_2^{18}O$.

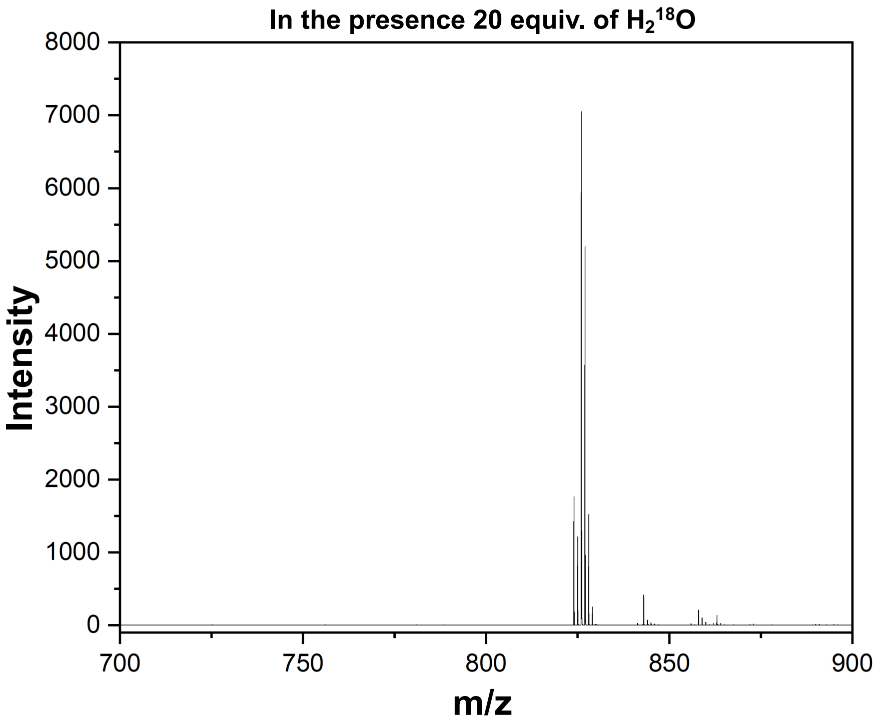

Supplementary Figure 28. Full HRMS of the reaction of 1a in the presence of 20.0 equiv. $H_2^{18}O$.

8. Supplementary references

1. (a) Hohenberg, P., Kohn, W. *Phys. Rev.* **136**, B864 (1964); (b) Kohn W., Sham, L. J. *Phys. Rev.*, **140**, A1133 (1965).

2. Gaussian 09, Revision D.01, M. J. Frisch, G. W. Trucks, H. B. Schlegel, G. E. Scuseria, M. A. Robb, J. R. Cheeseman, G. Scalmani, V. Barone, G. A. Petersson, H. Nakatsuji, X. Li, M. Caricato, A. Marenich, J. Bloino, B. G. Janesko, R. Gomperts, B. Mennucci, H. P. Hratchian, J. V. Ortiz, A. F. Izmaylov, J. L. Sonnenberg, D. Williams-Young, F. Ding, F. Lipparini, F. Egidi, J. Goings, B. Peng, A. Petrone, T. Henderson, D. Ranasinghe, V. G. Zakrzewski, J. Gao, N. Rega, G. Zheng, W. Liang, M. Hada, M. Ehara, K. Toyota, R. Fukuda, J. Hasegawa, M. Ishida, T. Nakajima, Y. Honda, O. Kitao, H. Nakai, T. Vreven, K. Throssell, J. A. Montgomery, Jr., J. E. Peralta, F. Ogliaro, M. Bearpark, J. J. Heyd, E. Brothers, K. N. Kudin, V. N. Staroverov, T. Keith, R. Kobayashi, J. Normand, K. Raghavachari, A. Rendell, J. C. Burant, S. S. Iyengar, J. Tomasi, M. Cossi, J. M. Millam, M. Klene, C. Adamo, R. Cammi, J. W. Ochterski, R. L. Martin, K. Morokuma, O. Farkas, J. B. Foresman, and D. J. Fox, *Gaussian, Inc.*, Wallingford CT, 2016.

3. Becke, A. D. *J. Chem. Phys.* **98**, 5648 (1993).

4. Peng, C., Schlegel, H. B. *Israel J. Chem.* **33**, 449 (1993).

5. Lin, H.-S., Jeon, I., Chen, Y., Yang, X.-Y., Nakagawa, T., Maruyama, S., Manzhos, S., Matsuo, Y. *Chem. Mater.* **31**, 8432 (2019).

6. Wong, W.-Y., Wang, X.-Z., He, Z.; Djurisic, A. B., Yip, C.-T., Cheung, K. Y., Wang, H., Mak, C. S. K., Chan, W.-K. *Nat. Mater.* **6**, 521 (2007).

7. Lin, H.-S., Jeon, I., Chen, Y., Yang, X.-Y., Nakagawa, T., Maruyama, S., Manzhos, S., Matsuo, Y. *Chem. Mater.* **31**, 8432 (2019).

8. Niu, C., Zhou, D.-B., Yang, Y., Yin, Z.-C. Wang, G.-W. *Chem. Sci.* 10, 3012–3017 (2019).
